# The Effects of Mild Intermittent Hypoxia Exposure on the Abdominal Subcutaneous Adipose Tissue Proteome in Overweight and Obese Men: A First-in-Human Randomized, Single-Blind, and Cross-Over Study

**DOI:** 10.3389/fphys.2021.791588

**Published:** 2022-01-04

**Authors:** Rens L. J. Van Meijel, Ping Wang, Freek Bouwman, Ellen E. Blaak, Edwin C. M. Mariman, Gijs H. Goossens

**Affiliations:** ^1^Department of Human Biology, NUTRIM School of Nutrition and Translational Research in Metabolism, Maastricht University Medical Center^+^, Maastricht, Netherlands; ^2^Department of Clinical Genetics, Maastricht University Medical Center^+^, Maastricht, Netherlands

**Keywords:** adipose tissue, proteomics, mild intermittent hypoxia, obesity, RCT

## Abstract

Adipose tissue (AT) oxygen tension (pO_2_) has been implicated in AT dysfunction and metabolic perturbations in both rodents and humans. Compelling evidence suggests that hypoxia exposure alters metabolism, at least partly through effects on AT. However, it remains to be elucidated whether mild intermittent hypoxia (MIH) exposure impacts the AT proteome. We performed a randomized, single-blind, and cross-over study to investigate the effects of seven consecutive days of MIH (FiO_2_ 15%, 3x2h/d) compared to normoxia (FiO_2_ 21%) exposure on the AT proteome in overweight/obese men. *In vivo* AT insulin sensitivity was determined by the gold standard hyperinsulinemic-euglycemic clamp, and abdominal subcutaneous AT biopsies were collected under normoxic fasting conditions following both exposure regimens (day 8). AT proteins were isolated and quantified using liquid chromatography-mass spectrometry. After correction for blood contamination, 1,022 AT protein IDs were identified, of which 123 were differentially expressed following MIH (*p* < 0.05). We demonstrate for the first time that MIH exposure, which markedly reduces *in vivo* AT oxygen tension, impacts the human AT proteome. Although we cannot exclude that a single differentially expressed protein might be a false positive finding, several functional pathways were altered by MIH exposure, also after adjustment for multiple testing. Specifically, differentially expressed proteins were involved in redox systems, cell-adhesion, actin cytoskeleton organization, extracellular matrix composition, and energy metabolism. The MIH-induced change in AT TMOD3 expression was strongly related to altered *in vivo* AT insulin sensitivity, thus linking MIH-induced effects on the AT proteome to metabolic changes in overweight/obese humans.

## Introduction

The prevalence of obesity has increased drastically over the last decades, with nearly a third of the world’s population living with overweight or obesity (Chooi et al., 2019). Obesity is a multifactorial disease, which is characterized by excess adipose tissue (AT) mass. AT is a metabolically active, endocrine organ, playing a central role in immunity, glucose and lipid homeostasis, angiogenesis, coagulation, vascular function, appetite regulation, and body weight control (Coelho et al., 2013). Thus, AT dysfunction is closely associated with an increased risk of cardiometabolic complications (Goossens, 2008, 2017; Rosen and Spiegelman, 2014) and hence mortality (Kopelman, 2000).

AT oxygen tension (pO_2_) has been implicated in AT dysfunction in both rodents and humans, as reviewed recently (Lempesis et al., 2020). Although AT hypoxia has been consistently shown in rodent models of obesity, conflicting findings have been reported in humans (Lempesis et al., 2020). We have previously demonstrated higher AT pO_2_ in obese compared to lean individuals (Goossens et al., 2011), decreased AT pO_2_ following diet-induced weight loss (Vink et al., 2017), and a positive association between AT pO_2_ and insulin resistance in humans, independently of adiposity (Goossens et al., 2018). Many *in vitro* studies have been performed to investigate whether exposure to hypoxic environments affects AT glucose and lipid metabolism. Indeed, hypoxia exposure appears to have profound effects on mRNA expression of several genes related to glucose and lipid metabolism in murine and human adipocytes, as reviewed extensively (Trayhurn, 2013; Lempesis et al., 2020). Importantly, the physiological effects of hypoxia are largely dependent upon the severity (mild versus severe), frequency (chronic versus intermittent), and duration (short versus long-term) of exposure (Navarrete-Opazo and Mitchell, 2014; Lempesis et al., 2020).

However, to investigate and better understand the effects of hypoxia exposure on a more functional level, proteomics analysis seems highly valuable. Indeed, previous studies have indicated that the proteomic profile of *3 T3-L1* adipocytes during long-term mild hypoxia exposure, at physiological pericellular oxygen concentrations (4% O_2_), affects various pathways involved in energy metabolism, suggestive of increased glycolytic metabolism and triacylglycerol synthesis (Weiszenstein et al., 2016). In addition, 24 h exposure to 1% O_2_ mainly increased expression of proteins related to the extracellular matrix (ECM) in human adipose stem cells (Riis et al., 2016). These *in vitro* findings suggest that hypoxia may induce ECM remodeling and induce metabolic changes in AT. Importantly, however, human *in vivo* studies that examined the effects of prolonged mild intermittent hypoxia (MIH) exposure on the AT proteome are lacking.

In the present study, we investigated the impact of MIH compared to normoxia exposure on the abdominal subcutaneous AT proteome in overweight and obese men, using untargeted liquid chromatography-mass spectrometry, to elucidate the physiological and functional adaptations in human AT evoked by MIH. Secondly, we explored the associations between MIH-induced alterations in AT protein expression and *in vivo* AT insulin sensitivity.

## Materials and Methods

### Subjects

Twelve overweight and obese (BMI ≥28 kg/m^2^) male subjects were recruited to participate in the present study. Subjects also needed to be aged between 30 and 65 years and insulin resistant, defined as HOMA-IR index ≥2.2. Exclusion criteria were smoking, cardiovascular disease, type 2 diabetes mellitus, liver or kidney malfunction, use of medication known to affect body weight and glucose metabolism, and marked alcohol consumption (>14 alcoholic units/wk). Furthermore, subjects had to be weight stable (weight change <3.0 kg) for at least three months prior to the start of the study. Participants were asked to refrain from drinking alcohol and perform no exercise 24 h prior to the start and during exposure regimen. The study, registered at Netherlands Trial Register (NL7120/NTR7325), was performed according to the Declaration of Helsinki and was approved by the Medical-Ethical Committee of Maastricht University. All subjects gave their written informed consent before participation in the study.

### Study Design

The present study was part of a randomized, double-blind, and placebo-controlled cross-over trial designed to investigate the metabolic effects of MIH in humans (van Meijel et al., 2021). Briefly, participants enrolled in this randomized, single-blind, and cross-over study were exposed to normobaric MIH (15% FiO_2_) and normobaric normoxia (21% FiO_2_) for 7 consecutive days (3 cycles of 2 h/d with 1 h of normoxia exposure between hypoxic cycles), separated by a 3–6 weeks. wash-out period. As described previously, hypoxia exposure was performed in an in-house manufactured airtight clinical room with the capability to accurately adjust oxygen availability at the Metabolic Research Unit Maastricht (Maastricht University, Netherlands). The oxygen level was set and maintained at 15.0 ± 0.2% for the hypoxia exposure regimen. Participants were blinded for the exposure regimen (hypoxia or normoxia; van Meijel et al., 2021). Systemic oxygen saturation was continuously monitored throughout the exposure regimens by finger pulse oximetry (Nellcor N-595 Pulse oximeter, Nellcor). At day 6 of the exposure regimens, AT pO_2_ was determined using an optochemical measurement system for continuous monitoring of tissue pO_2_, as described previously (Goossens et al., 2011). Both systemic oxygen saturation and AT pO_2_ were determined during hypoxia (or normoxia as control) exposure. At day 8, following the 7-day MIH/normoxia exposure regimens, fasting abdominal subcutaneous adipose tissue biopsies were collected, and a two-step hyperinsulinemic-euglycemic clamp was performed to determine AT, hepatic, and peripheral insulin sensitivity under normoxic conditions, as described previously (Reijnders et al., 2016).

### Abdominal Subcutaneous Adipose Tissue Biopsy

Fasting abdominal subcutaneous AT biopsies were collected (approximately 1 g) using needle aspiration under local anesthesia (2% lidocaine), 6–8 cm lateral from the umbilicus. After thorough rinsing with sterile saline, visible blood vessels were removed using sterile tweezers. Subsequently, specimens were snap-frozen using liquid nitrogen and stored at −80°C for further analysis. Due to sampling issues, we could not collect enough material following hypoxia exposure in one participant. Therefore, paired adipose tissue biopsies (following both MIH and normoxia exposure) were available for analysis in *n* = 11 individuals.

### Protein Isolation and Preparation for LC-MS

Frozen AT (~100 mg) was ground in a mortar with liquid nitrogen. Per microgram of grounded powder, 2 μl of 50  mm ammonium bicarbonate with 5 M urea, was added to dissolve the powder. The solution was freeze-thawed in liquid nitrogen 3 times after which it was vortexed for 5 min. The homogenate was centrifuged at 20,000 g for 30 min at 10°C. The supernatant was carefully collected and protein concentrations were determined with a Bradford-based protein assay (Bio-Rad, Veenendaal, Netherlands).

A total of 100 μg protein in 50 μl 50 mm ammonium bicarbonate (ABC) with 5 M urea was used. 5 μl of DTT solution (20 mm final) was added and incubated at room temperature for 45 min. The proteins were alkylated by adding 6 μl of IAA solution (40 mm final). The reaction was taken place at room temperature for 45 min in the darkness. The alkylation was stopped by adding 10 μl of DTT solution (to consume any unreacted IAA) and incubated at room temperature for 45 min. For the digestion, 2 μg trypsin/lysC was added to the protein and incubated at 37°C for 2 h. 200 μl of 50 mm ABC was added to dilute the urea concentration and further incubate at 37°C for 18 h. The digestion mixture was centrifuged at 2.500 g for 5 min and the supernatant, which contained the peptide mixture, collected for the use of LCMS analysis.

### Protein Identification and Quantification Using LC-MS

A nanoflow HPLC instrument (Dionex ultimate 3,000) was coupled online to a Q Exactive (Thermo Scientific) with a nano-electrospray Flex ion source (Proxeon). Each sample was run separately for label free quantification. 5 μl of the peptide mixture was loaded into a C18-reversed phase column (Thermo Scientific, Acclaim PepMap C18 column, 75-μm inner diameter x 15 cm, and 2-μm particle size). The peptides were separated with a 240 min linear gradient of 4–50% in buffer A (100% water with 0.1% TFA) with buffer B (80% acetonitrile and 0.08% formic acid) at a flow rate of 300 nl/min. MS data were acquired using a data-dependent top-10 method, dynamically choosing the most abundant precursor ions from the survey scan (280–1,400 m/z) in positive mode. Survey scans were acquired at a resolution of 70,000 and a maximum injection time of 120 ms. Dynamic exclusion duration was 30 s. Isolation of precursors was performed with a 1.8 m/z window and a maximum injection time of 200 ms. Resolution for HCD spectra was set to 17,500 and the Normalized Collision Energy was 30 eV. The under-fill ratio was defined as 1.0%. The instrument was run with peptide recognition mode enabled, but exclusion of singly charged and charge states of more than five.

### Database Search and Quantification

The MS data were searched using Proteome Discoverer 2.2 Sequest HT search engine (Thermo Scientific), against the UniProt human database. The false discovery rate (FDR) was set to 0.01 for proteins and peptides, which had to have a minimum length of 6 amino acids. The precursor mass tolerance was set at 10 ppm and the fragment tolerance at 0.02 Da. One miss-cleavage was tolerated, oxidation of methionine was set as a dynamic modification. Carbamidomethylation of cysteines was set as fixed modifications. Label free quantitation was conducted using the Minora Feature Detector node in the processing step and the Feature Mapper node combined with the Precursor Ions Quantifier node in the consensus step with default settings within Proteome Discoverer 2.2 (Qiao et al., 2019).

### Protein Signal Normalization and Adjustment for Blood Protein Contamination

The inter-run variation was normalized using ppm fractional normalization. To reduce the influence of the blood protein contamination on the AT proteome, we retrieved information from the UniProt database and GeneCards to set up a blood protein exclusion list with known proteins exclusively expressed in blood, including all immunoglobulins (Anderson and Anderson, 2002; Stelzer et al., 2016). The amount of signal contributed by these blood proteins in our AT sample were found with a mean value of 76%. We defined valid human AT proteins based on these quality criteria: (1) not exclusively expressed in blood, (2) identification Score Sequest HT ≥ 2.5, 3 present in >50% (≥6 of 11) samples in at least one treatment group. The final signal of a valid human AT protein was the protein relative ppm abundance in a fixed amount (76%) blood contaminated AT sample. This final signal (below referred to as “signal”) was used in data analysis (see equation).


$$\begin{array}{l} \mathrm{Final signal}\;\left[\mathrm{protein}\;x,\;\mathrm{sample}\;i\right]\\ =\frac{\mathrm{Normalized signal}\;\left[\mathrm{protein}\;x,\;\mathrm{sample}\;i\right]}{\sum \mathrm{norm}\mathrm{.signal}\;\left[\mathrm{sample}\;i\right]-\sum \mathrm{Blood prot}\mathrm{.norm}\mathrm{.signal}\;\left[\mathrm{sample}\;i\right]}\times 10^{6}\times \left(1-0.76\right) \end{array}$$

### Statistical Analysis

Only valid human AT proteins were taken into statistical analyses. First, missing values (9.5% of total number of data points) were imputed using random forest algorithm with R missForest package v1.4. Thereafter, data were log2 transformed. The effects of MIH compared to normoxia exposure on abdominal subcutaneous AT protein expression were assessed using two-sided paired Student’s t-tests. The fold change was calculated using the back-transformed means for both conditions. The Benjamini-Hochberg procedure was applied to control for multiple testing, with false discovery rate set at 0.25, and q-values were calculated. Proteins with a *value of p* < 0.05 (MIH versus normoxia exposure) were selected for further biological annotation and analysis. Subsequently, these differentially expressed proteins (*p* < 0.05) were imported into Cytoscape plug-in ClueGO v2.5.7 for functional analysis (Bindea et al., 2009), based on GO biological process terms and KEGG pathways dated on 08.05.2020. For this functional analysis, Bonferroni’s step-down method was applied, and only functional groups with adjusted value of *p* < 0.05 were considered as significantly affected by MIH compared to normoxia exposure. Spearman’s rank correlation analysis was performed to determine correlations between changes in all 123 differentially expressed AT proteins and changes in AT insulin sensitivity [expressed as suppression (%) of FFA plasma concentration upon 10 mU∙m^−2^ insulin infusion], with subsequent multiple testing correction (Benjamini-Hochberg Procedure). All statistical analyses were performed in R environment, version 3.5, with various packages (stats, missForest, gplots and pheatmap) and SPSS.

## Results

Characteristics of the study participants are shown in Table 1. All individuals (age range 52–65 yrs) were overweight or obese (BMI ≥ 28 kg∙m^−2^) and demonstrated a HOMA-IR index ≥2.2.

**Table 1:** Baseline characteristics of male study participants.

	Baseline
Age (y)	61 ± 1
BMI (kg/m^2^)	30.8 ± 3.6
Hemoglobin (mmol•l^−1^)	9.5 ± 0.5
HbA1c (%)	5.6 ± 0.1
Fasting glucose (mmol•l^−1^)	5.7 ± 0.5
2 h-glucose (mmol•l^−1^)	6.2 ± 1.3
HOMA-IR	3.7 ± 0.4

*Hb, hemoglobin; Hb1Ac, glycated hemoglobin; HOMA-IR, homeostasis model of assessment of insulin resistance; and 2 h-glucose determined during a 75 g oral glucose tolerance test (OGTT). Values are mean ± SEM (n = 11)*.

### Mild Intermittent Hypoxia Exposure Decreases Adipose Tissue Oxygen Tension

To provide the proof-of-concept in humans that MIH exposure reduces oxygen availability in abdominal subcutaneous AT, we determined both systemic oxygen saturation (finger pulse oximetry, measured at days 1–5) and AT pO_2_ (measured at day 6), using a highly accurate, microdialysis-based optochemical measurement system to continuously monitor AT pO_2_ *in vivo* in humans (Goossens et al., 2011). As previously reported (van Meijel et al., 2021), MIH exposure reduced systemic oxygen saturation (normoxia: 97.1 ± 0.3 vs. hypoxia: 92.0 ± 0.5%, *p* < 0.001) and decreased AT pO_2_ (normoxia: 36.5 ± 1.5 mmHg versus hypoxia: 21.0 ± 2.3 mmHg, *p* < 0.001).

### Mild Intermittent Hypoxia Exposure Impacts the Adipose Tissue Proteome

**Figure 1:**
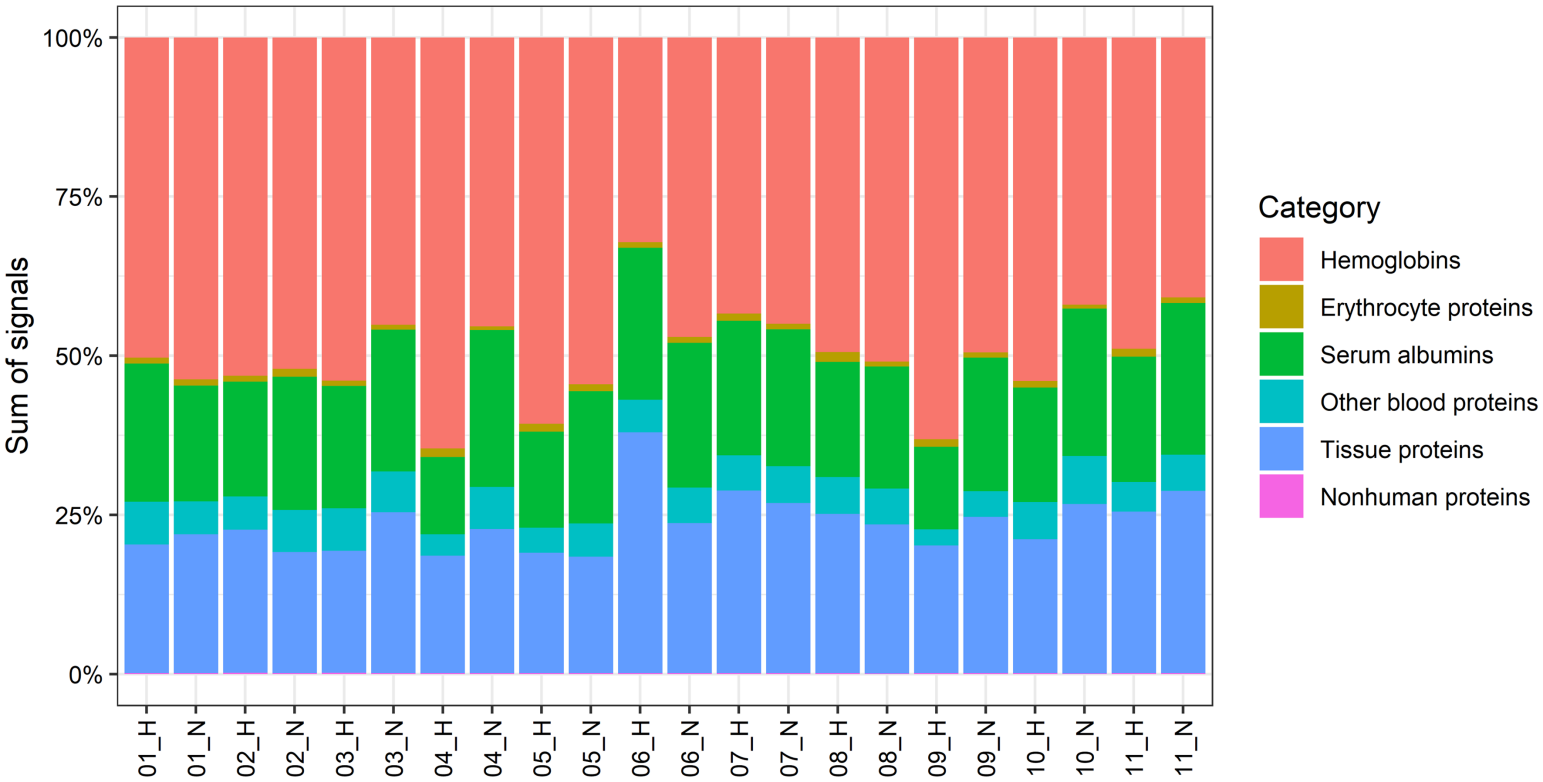
Contribution to the total proteome signals of AT by human abdominal subcutaneous adipose tissue proteins and different groups of blood-specific proteins. Each bar represents an adipose tissue biopsy, with number indicating the respective participant. H; biopsy after mild intermittent hypoxia (MIH) exposure; N; normoxia exposure.

**Figure 2:**
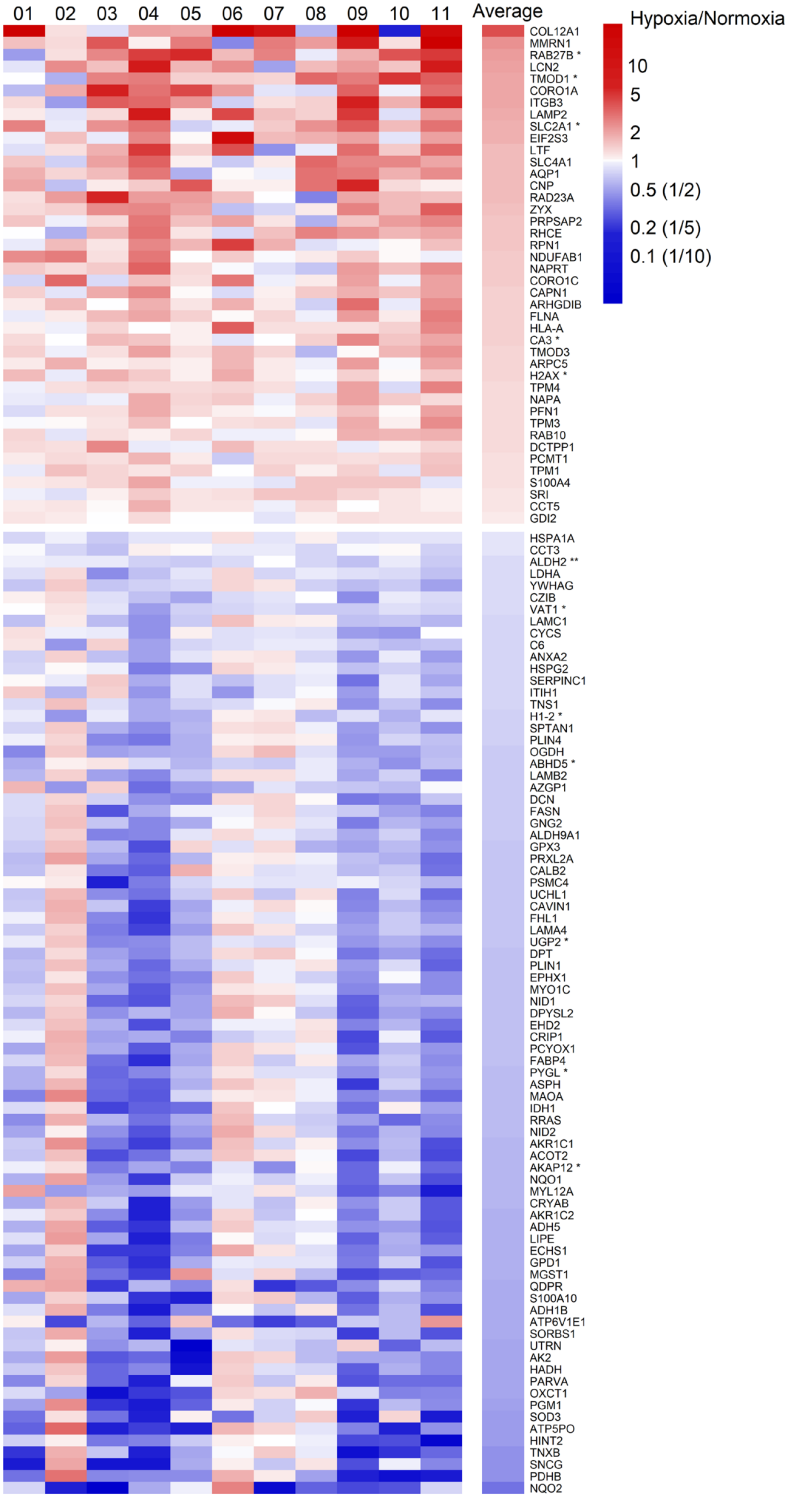
Heatmap of fold changes of differentially expressed proteins at a significance level of *p* < 0.05 in abdominal subcutaneous adipose tissue following MIH compared to normoxia exposure in overweight and obese humans (*n* = 11). These proteins are labeled by gene symbols and are sorted by average fold changes. Each column represents one individual participant labeled by number, and the last column shows the average fold change. The color key is proportional to the log2 transformed fold change. ^*^p < 0.01; ^**^p < 0.001.

We quantified 1,091 accession IDs of 1,074 proteins in the AT samples with HT score > 2.5, which appeared in more than half of the samples in at least one of the conditions (MIH and/or normoxia). Several of these proteins were considered blood-specific (e.g., hemoglobins, serum albumins, and erythrocyte proteins), explained by AT specimen contamination with some blood during sample collection, despite thorough cleaning using sterile saline, as described previously (Vogel et al., 2019). Indeed, 69 identified IDs of 63 unique proteins were blood-specific and contributed to 62–82% of the total protein signal (Figure 1). Therefore, we corrected the quantification after removal of these blood-specific proteins (Supplementary Table 1). In the resulting 1,022 AT protein IDs for 1,011 proteins, 123 IDs of 123 unique proteins were differentially expressed (*p* < 0.05; Table 2; Supplementary Table 2) following MIH compared to normoxia exposure (42 upregulated and 81 downregulated), as visualized in a heat map (Figure 2). Differential expression of top 5 upregulated/downregulated (based on *value of p*s) proteins per individual is visualized in Supplementary Figure 1. Changes of individual proteins did not reach statistical significance after controlling for multiple testing (false discovery rate set at 0.25).

**Table 2:** Top 20 up- and downregulated proteins by MIH compared to normoxia exposure in overweight and obese individuals.

Upregulated	Uniprot	Gene	Protein names	Fold change (Hypoxia/Normoxia)	*value of p*	*q*-value
1	P11166	SLC2A1	Solute carrier family 2, facilitated glucose transporter member 1 (Glucose transporter type 1)	1.81	0.006	0.387
2	P28289	TMOD1	Tropomodulin-1 (Erythrocyte tropomodulin)	1.97	0.006	0.387
3	P16104	H2AX	Histone H2AX	1.29	0.007	0.387
4	O00194	RAB27B	Ras-related protein Rab-27B	2.16	0.008	0.387
5	P07451	CA3	Carbonic anhydrase 3	1.36	0.009	0.387
6	P29972	AQP1	Aquaporin-1 (Water channel protein for red blood cells and kidney proximal tubule)	1.63	0.013	0.387
7	P07384	CAPN1	Calpain-1 catalytic subunit	1.39	0.013	0.387
8	P02730	SLC4A1	Band 3 anion transport protein	1.65	0.015	0.387
9	P22061	PCMT1	Protein-L-isoaspartate(D-aspartate) O-methyltransferase	1.21	0.015	0.387
10	P21333	FLNA	Filamin-A	1.38	0.015	0.387
11	O15511	ARPC5	Actin-related protein 2/3 complex subunit 5	1.30	0.015	0.387
12	P09493	TPM1	Tropomyosin alpha-1 chain	1.21	0.016	0.387
13	P31146	CORO1A	Coronin-1A	1.93	0.018	0.387
14	Q15942	ZYX	Zyxin	1.59	0.018	0.387
15	O60256	PRPSAP2	Phosphoribosyl pyrophosphate synthase-associated protein 2	1.50	0.018	0.387
16	P80188	LCN2	Neutrophil gelatinase-associated lipocalin	2.04	0.018	0.387
17	P48643	CCT5	T-complex protein 1 subunit epsilon	1.15	0.021	0.387
18	O14561	NDUFAB1	Acyl carrier protein, mitochondrial	1.43	0.022	0.387
19	P30626	SRI	Sorcin	1.17	0.022	0.387
20	P13473	LAMP2	Lysosome-associated membrane glycoprotein 2	1.85	0.025	0.387
**Downregulated**	**Uniprot**	**Gene**	**Protein names**	**Fold change (Hypoxia/Normoxia)**	***value of p***	***q-value***
1	P05091	ALDH2	Aldehyde dehydrogenase, mitochondrial	0.81	8.7E-04	0.387
2	Q8WTS1	ABHD5	1-acylglycerol-3-phosphate O-acyltransferase ABHD5 (Lipid droplet-binding protein CGI-58)	0.70	0.003	0.387
3	Q99536	VAT1	Synaptic vesicle membrane protein VAT-1 homolog	0.78	0.004	0.387
4	P06737	PYGL	Glycogen phosphorylase, liver form	0.62	0.006	0.387
5	Q02952	AKAP12	A-kinase anchor protein 12	0.59	0.008	0.387
6	Q16851	UGP2	UTP-glucose-1-phosphate uridylyltransferase	0.65	0.008	0.387
7	P16403	H1-2	Histone H1.2	0.73	0.009	0.387
8	P07099	EPHX1	Epoxide hydrolase 1	0.64	0.014	0.387
9	P16083	NQO2	Ribosyldihydronicotinamide dehydrogenase [quinone]	0.33	0.014	0.387
10	Q9NVD7	PARVA	Alpha-parvin	0.51	0.015	0.387
11	P21695	GPD1	Glycerol-3-phosphate dehydrogenase [NAD(+)], cytoplasmic	0.56	0.016	0.387
12	P02511	CRYAB	Alpha-crystallin B chain	0.58	0.017	0.387
13	P10301	RRAS	Ras-related protein R-Ras	0.61	0.017	0.387
14	Q16836	HADH	Hydroxyacyl-coenzyme A dehydrogenase, mitochondrial	0.51	0.018	0.387
15	O60240	PLIN1	Perilipin-1 (Lipid droplet-associated protein)	0.65	0.019	0.387
16	Q9BX66	SORBS1	Sorbin and SH3 domain-containing protein 1	0.53	0.019	0.387
17	Q14112	NID2	Nidogen-2	0.60	0.019	0.387
18	P08294	SOD3	Extracellular superoxide dismutase	0.47	0.020	0.387
19	P36871	PGM1	Phosphoglucomutase-1	0.48	0.020	0.387
20	Q9BX68	HINT2	Histidine triad nucleotide-binding protein 2, mitochondrial	0.46	0.021	0.387

### Mild Intermittent Hypoxia Exposure Alters Adipose Tissue Expression of Proteins Involved in Structural and Metabolic Pathways

**Figure 3:**
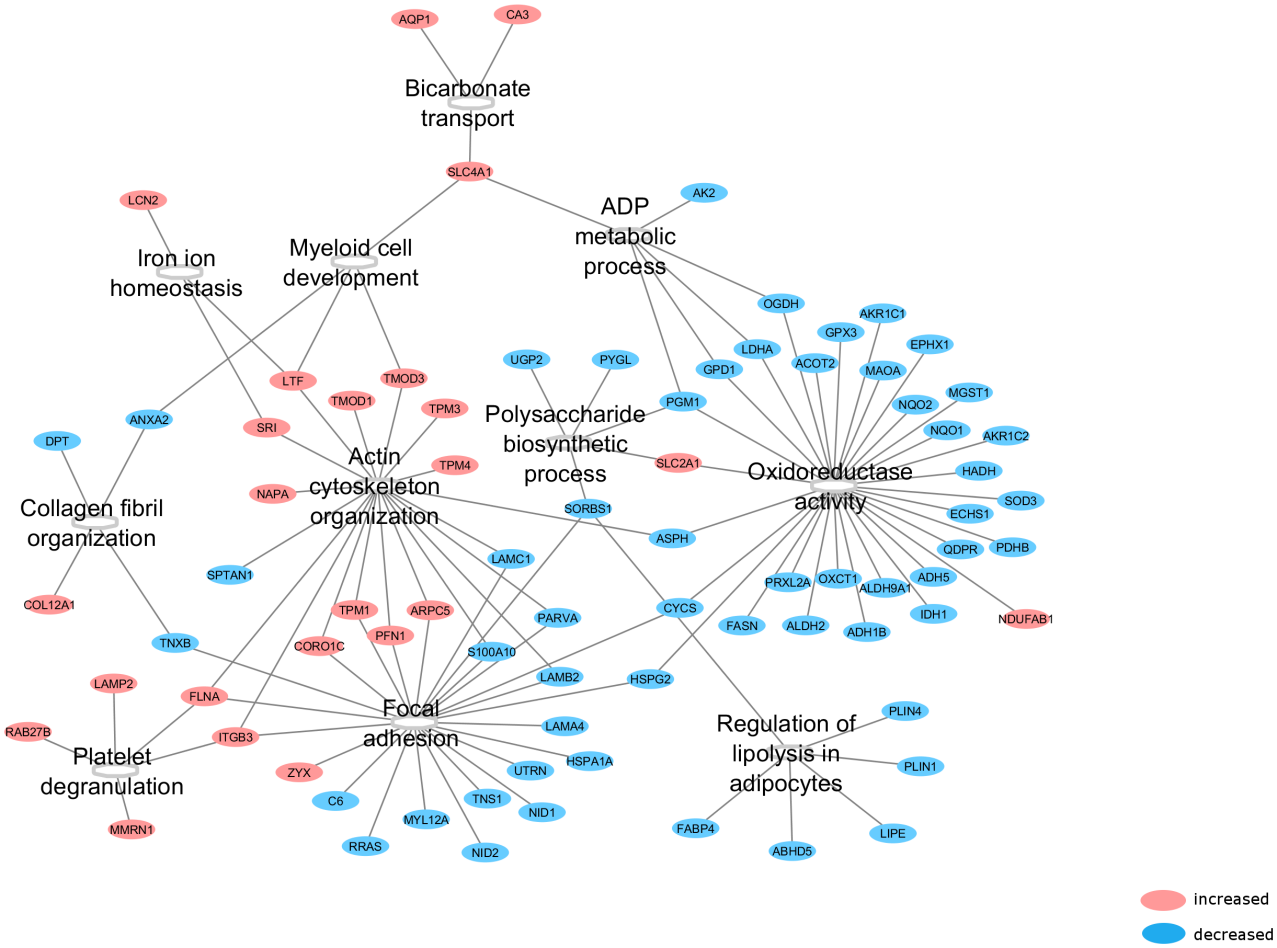
Functional groups affected by MIH exposure. After functional annotation of differentially expressed proteins, 11 functional groups were identified (*p* < 0.05, corrected with Bonferroni step-down procedure) with associated proteins which were altered by MIH exposure. Red encircled proteins represent upregulated, whereas blue encircled proteins represent downregulated proteins subsequent to MIH exposure.

All 123 differentially expressed proteins were functionally annotated, since MIH-induced changes in specific functional pathways provide more robust information related to functional changes than alterations in single proteins. Thus, 11 functional groups that were enriched in these differentially expressed proteins were identified (Figure 3). These functional groups cover 104 representative GO terms and pathways (Supplementary Table 3), to which 79 proteins are associated (Figure 3). The functional groups cover predominantly structural and metabolic-related GO terms and pathways. MIH-induced AT expression of several proteins related to actin cytoskeleton organization, focal adhesion, and myeloid development, whereas it reduced the expression of proteins related to collagen fibril organization (Table 3; Figure 3). In addition, MIH reduced several pathways related to oxidoreductase activity, regulation of lipolysis in adipocytes, polysaccharide biosynthetic, and ADP metabolic processes. Moreover, MIH increased proteins involved in bicarbonate transport, iron ion homeostasis, as well as platelet degranulation (Table 3; Figure 3).

**Table 3:** Functional groups affected by mild intermittent hypoxia based on representative GO terms and KEGG pathways.

Functional group	Group adjusted *value of p*
Oxidoreductase activity	4.7E-10
Focal adhesion	2.8E-09
Actin cytoskeleton organization	1.0E-06
Collagen fibril organization	0.003
ADP metabolic process	0.005
Myeloid cell development	0.009
Bicarbonate transport	0.010
Platelet degranulation	0.010
Regulation of lipolysis in adipocytes	0.010
Iron ion homeostasis	0.012
Polysaccharide biosynthetic process	0.012

*Bonferroni step-down corrected p-values. GO, Gene Ontology; KEGG, Kyoto Encyclopedia for Genes and Genomes*.

### Mild Intermittent Hypoxia-Induced Effects on the Adipose Tissue Proteome Are Related to *in vivo* Adipose Tissue Insulin Sensitivity

**Figure 4:**
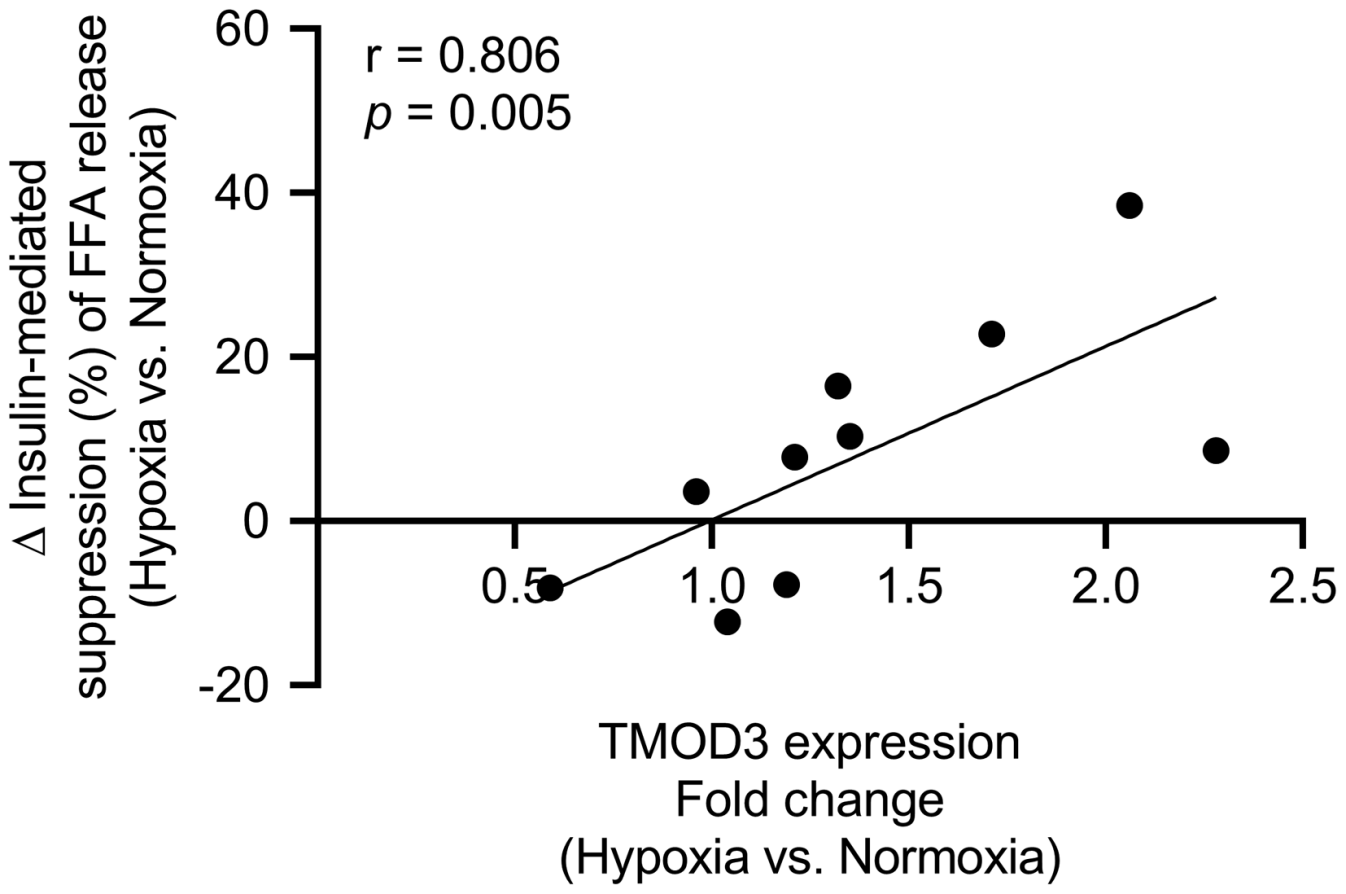
Association between TMOD3 and adipose tissue insulin sensitivity [insulin-mediated suppression of plasma free fatty acids (%)]. The MIH-induced increase in TMOD3 expression was correlated with improved adipose tissue insulin sensitivity (*n* = 10). TMOD3; tropomodulin-3.

Since MIH exposure had a significant impact on several ECM- and cytoskeleton-related proteins, as well as proteins involved in energy metabolism, we next determined the associations between differentially expressed AT proteins and *in vivo* AT insulin sensitivity. Although AT insulin sensitivity did not significantly change following MIH exposure (data not shown), the increase in tropomodulin-3 (TMOD3) protein expression evoked by MIH exposure was positively associated with the change in AT insulin sensitivity (*r* = 0.806 *p* = 0.005, Figure 4). However, after multiple testing correction, this correlation did not remain statistically significant (*q* = 0.615).

## Discussion

In the present randomized, single-blind, and cross-over study, we examined for the first time the effects of 7 consecutive days of MIH compared to normoxia exposure on the abdominal subcutaneous AT proteome in overweight and obese men. From the 1,011 AT-specific proteins identified, 123 proteins were differentially expressed following MIH compared to normoxia exposure. Specifically, MIH-induced marked alterations of functional groups, mainly related to oxidoreductase systems, cell-adhesion, actin cytoskeleton and ECM organization, and energy metabolism. Moreover, the MIH-induced increase in TMOD3 expression was significantly related to improved AT insulin sensitivity, suggesting a link between MIH-induced effects on the AT proteome and metabolic changes in human AT. Although we cannot exclude that a single differentially expressed protein might be a false positive finding, the fact that these functional pathways (consisting of many proteins) were significantly upregulated/downregulated after multiple testing correction and with high concordance between its protein members provides strong evidence that MIH exposure alters specific protein pathways within the adipose tissue proteome.

We found that MIH exposure significantly downregulates oxidoreductase activity-related pathways in AT. More specifically, NAD(P)- and NAD(P)H-dependent dehydrogenase activity-related pathways contributed to the changes in this functional group. Out of the 31 proteins associated with oxidoreductase activity, aldehyde dehydrogenase 2 (ALDH2), glycerol-3-phosphate dehydrogenase 1 (GPD1), and 3-hydroxyacyl-CoA dehydrogenase (HADH) were most significantly downregulated. This is in agreement with the MIH-induced reduction in AT gene expression of aldo-keto reductase family 7, member A2 (van Meijel et al., 2021). The expression of ALDH2, which catalyzes the oxidation of aldehydes, has been found to be increased with adiposity (Frohnert et al., 2011). ALDH2 may counteract reactive oxygen species (ROS)-induced lipid aldehyde formation, which has been described in obesity and insulin resistance (Pillon and Soulage, 2012). HADH, a key enzyme involved in fatty acid oxidation, was also downregulated by MIH exposure. In agreement with our findings, it has recently been shown that exposure to severe hypoxia exposure markedly reduced fatty acid oxidation in murine adipocytes (Lu et al., 2016). Of note, hypoxia decreases tissue NAD^+^ content and substantially increases the NADH/NAD+ ratio (Yurkov and Safonova, 1976; Eales et al., 2016). Most of these oxidoreductases are dependent upon NAD^+^ as an electron acceptor. As such, this may explain reduced expression of these enzymes following prolonged *in vivo* MIH exposure. Notably, an increased NADH/NAD^+^ ratio inhibits NAD + -dependent processes (Eales et al., 2016), covering a large number of central metabolic pathways, such as the tricarboxylic acid cycle and fatty acid catabolism (Supplementary Table 3). Taken together, our findings suggest that MIH reduced AT protein expression of oxidoreductases, thereby affecting the direction of redox processes, and may promote alternative pathways to yield energy under hypoxic conditions.

In addition, AT expression of several proteins related to focal adhesion-related processes was increased subsequent to MIH exposure. Focal adhesion between the basement membrane or the extracellular matrix (ECM) and the adipocytes regulates cytoskeletal changes as well as adipocyte differentiation (Kim and Yoo, 2015). In addition, focal adhesions exert a key role in ECM-mediated integrin signaling and may promote adipocyte survival and insulin sensitivity through focal adhesion kinase activity (Luk et al., 2017). Out of the 21 proteins associated with focal adhesion, zyxin (ZYX) was upregulated. ZYX is essential in localizing the focal adhesion sites and stress fibers generated by mechanical cues, thereby regulating the adipocyte cytoskeleton (Zhang et al., 2017). On the other hand, Ras-related (RRAS), which has been demonstrated to markedly enhance focal adhesion formation (Kwong et al., 2003), was most significantly downregulated. In accordance with these MIH-induced changes in proteins related to focal adhesion, we have previously found that MIH decreased the expression of genes related to focal adhesion in AT(van Meijel et al., 2021). Furthermore, several reports have demonstrated predominant effects of hypoxia on cytoskeletal organization *in vitro*, in particular an increase in actin-stress fibers in various cell types (Vogler et al., 2013; Gilkes et al., 2014).

In line with the above, MIH exposure induced the expression of processes related to actin cytoskeleton organization. Indeed, it has been suggested that actin remodeling is essential during adipocyte maturation, demonstrated by major changes in actin dynamics during adipocyte expansion (Hansson et al., 2019). Furthermore, actin remodeling might enhance insulin signaling and glucose transporter-4 (GLUT4) translocation, thus improving glucose homeostasis (Chiu et al., 2010; Hansson et al., 2019). Here, we found increased AT protein expression of actin-related protein 2/3 complex subunit 5 (ARPC5) and tropomodulin-1 (TMOD1), which are involved in the regulation of actin polymerization and differentiation (Yang et al., 2014). This is in line with recent evidence of MIH-induced upregulation of gene expression of the actin dynamics pathway in human AT(van Meijel et al., 2021). Furthermore, MIH exposure increased TMOD3 protein expression, which is essential for insulin signal transduction (Lim et al., 2015). Interestingly, the MIH-induced increase in AT TMOD3 expression was correlated with improved AT insulin sensitivity, although this correlation did not remain statistically significant after multiple testing correction. In agreement with this, we found that the expression of α-II spectrin (SPTAN1) in AT, which appeared to be higher in subcutaneous adipocytes of obese insulin resistant compared to insulin sensitive individuals (Xie et al., 2016), was reduced after MIH exposure. Collectively, these findings suggest that prolonged exposure to MIH has pronounced effects on dynamics of focal adhesion, as well as AT remodeling, which may contribute to improved AT insulin sensitivity.

Interestingly, we found that collagen fibril organization was reduced by MIH exposure. It has previously been suggested that hypoxia induces AT fibrosis, by increasing collagen type I deposition, resulting in increased stiffness of the ECM (Buechler et al., 2015). The present findings, however, show that MIH reduced the expression of tenascin-X (TNXB) and dermatopontin (DPT), which are both implicated in collagen fibrillogenesis (Minamitani et al., 2004; Okamoto and Fujiwara, 2006). In line with these findings, we have previously found reduced expression of genes involved in assembly of collagen and other multimeric components in AT after MIH exposure (van Meijel et al., 2021). Of note, it has been demonstrated that circulating levels and visceral AT mRNA expression of DPT are increased in obese insulin-resistant individuals (Unamuno et al., 2020). Furthermore, we found 4-fold increased AT protein expression of collagen type XII α 1 chain (COL12A1), which is a non-fibrillar type of collagen involved in adipocyte differentiation, and as such a putative marker of adipogenesis (Tahara et al., 2004). Taken together, our findings indicate that MIH may elicit ECM remodeling and reduce fibrillogenesis in AT, which may enhance the fat storage capacity of adipocytes, allowing safe storage of lipids in abdominal subcutaneous AT (Luk et al., 2017).

Furthermore, AT proteins associated with the regulation of lipolysis in adipocytes appeared to be reduced by MIH. Indeed, we found decreased expression of abhydrolase domain-containing 5 (ABDH5), fatty acid binding protein 4 (FABP4), hormone-sensitive lipase (LIPE) and perilipin-1 (PLIN1), and PLIN4 were found. ABDH5 and PLIN1, which appeared to be most significantly downregulated, exert an important role in lipid catabolism, acting as activator for triacylglycerol hydrolases and coating protein surrounding lipid droplets in adipocytes, respectively (Oberer et al., 2011; Sztalryd and Brasaemle, 2017). It has been reported that long-term hypoxia (5% O_2_) reduced ABDH5 and tended to reduce PLIN1 expression in *3 T3-L1* adipocytes compared to standard laboratory conditions (21% O_2_; Hashimoto et al., 2013). In agreement with our findings, reduced LIPE expression subsequent to long-term hypoxia exposure was also observed in that study (Hashimoto et al., 2013). In addition, it has been demonstrated that chronic hypoxia exposure (3% O_2_) may reduce lipid storage and mobilization in human (pre)adipocytes (Mahat et al., 2021). Thus, the present findings, together with results from previous *in vitro* studies, suggest that MIH exposure may reduce lipid turnover in human AT to adapt to the lower O_2_ availability. Moreover, this may imply that MIH exposure evokes a shift toward glycolytic metabolism to provide energy for the cells.

Indeed, MIH decreased the expression of several proteins associated with biosynthesis of polysaccharides, and therefore suggests that functional alterations in glycogen metabolism are associated with cellular adaptations to hypoxia, as previously described (Favaro et al., 2012). However, both UDP-glucose pyrophosphorylase 2 (UGP2), phosphoglucomutase 1 (PGM1), and glycogen phosphorylase l (PYGL) enzymes were downregulated subsequent to MIH exposure, suggestive of lower glycogen flux. In adipocytes, it has been demonstrated that hypoxia increases glycogen synthesis, which may induce autophagic flux (Ceperuelo-Mallafre et al., 2016). Furthermore, the present data also show a ~ 2-fold increase in AT expression of glucose transporter 1 (SLC2A1) protein after MIH exposure, which may imply that hypoxia enhances insulin-independent glucose uptake. Indeed, it has been demonstrated that hypoxic treatment induces insulin-independent glucose transport in various cell types, mainly mediated by SLC2A1 (Wood et al., 2007; Lu et al., 2016). Thus, the present findings suggest that MIH exposure alters glucose homeostasis within AT.

Next, we found that MIH induced several AT proteins associated with iron ion homeostasis. In line with the present results, (hypobaric) hypoxia exposure has previously been implicated in iron homeostasis, affecting the regulation of several enzymes involved in iron absorption, such as repression of hepcidin (Goetze et al., 2013; Shah and Xie, 2014). In addition, bicarbonate transport appeared to be upregulated by MIH, which has also been described previously in tumor hypoxia (McIntyre et al., 2016). The latter may be related to hypoxia-induced lactate production, thereby decreasing interstitial pH (Ye, 2009), which might explain increased bicarbonate transport subsequent to MIH.

Although we have filtered out proteins exclusively abundant in blood in our analysis, the present data analysis still covered some proteins enriched in blood, such as lactotransferrin (LTF), and the altered AT expression of these proteins may be due to blood cells. We found that MIH exposure increased the functional groups “platelet degranulation” and “myeloid cell development,” reflecting functional alterations in blood cells. In line, it has been shown that human platelets exposed to 5% O_2_ were characterized by altered phenotype and enhanced activity (Cameron et al., 2018). However, conflicting findings have also been reported in humans. Healthy individuals exposed to short-term severe hypoxia *in vivo* (8% O_2_) did not show any differences in platelet activity, and hence blood coagulation (Mantysaari et al., 2011). Therefore, the functional implications of MIH-induced alterations in the expression of proteins associated with platelet degranulation remain to be elucidated.

In conclusion, the present study demonstrates for the first time that human *in vivo* MIH exposure for seven consecutive days affects the abdominal subcutaneous AT proteome in overweight and obese men. Moreover, we found that the increased expression of TMOD3 was associated with improved AT insulin sensitivity following MIH exposure, thereby linking MIH-induced adaptations in the AT proteome to metabolic changes in human AT. Although we cannot exclude that altered expression of certain proteins might be due to false positive findings, the fact that changes in several functional pathways were found after multiple testing correction provides further evidence that MIH exposure impacts the human adipose tissue proteome. Further studies are needed to confirm the present findings, and to elucidate whether adipocytes and/or other cell types present in adipose tissue are responsible for the MIH-induced changes in the human AT proteome that we found in the present study.

## Data Availability Statement

The original contributions presented in the study are publicly available. This data can be found at: http://proteomecentral.proteomexchange.org/cgi/GetDataset?ID=PXD029213.

## Ethics Statement

The studies involving human participants were reviewed and approved by Medical-Ethical Committee of Maastricht University. The patients/participants provided their written informed consent to participate in this study.

## Author Contributions

RVM, EB, and GG designed the research. RVM and GG performed the sample collection. FB contributed to the sample preparation, data acquisition and the LCMS analysis. FB performed the LCMS analysis. PW and RVM performed the data analysis. RVM wrote the manuscript. PW, FB, EB, EM, and GG revised the manuscript. All authors contributed to data interpretation and approved the final version of the manuscript.

## Funding

This study was funded by a senior fellowship awarded to GG by the Dutch Diabetes Research Foundation (grant number: 2015.82.1818).

## Conflict of Interest

The authors declare that the research was conducted in the absence of any commercial or financial relationships that could be construed as a potential conflict of interest.

## Publisher’s Note

All claims expressed in this article are solely those of the authors and do not necessarily represent those of their affiliated organizations, or those of the publisher, the editors and the reviewers. Any product that may be evaluated in this article, or claim that may be made by its manufacturer, is not guaranteed or endorsed by the publisher.
