# Supplementary material for: The Effects of Mild Intermittent Hypoxia Exposure on the Abdominal Subcutaneous Adipose Tissue Proteome in Overweight and Obese Men: A First-in-Human Randomized, Single-Blind, and Cross-Over Study
Source: Front Physiol. 2022 Jan 4;12:791588. doi: 10.3389/fphys.2021.791588 (PMC8764283; doi:10.3389/fphys.2021.791588)
Supplement: Supplementary file 1 [file Data_Sheet_1.pdf]

Supplementary Material

1 Supplementary Figures

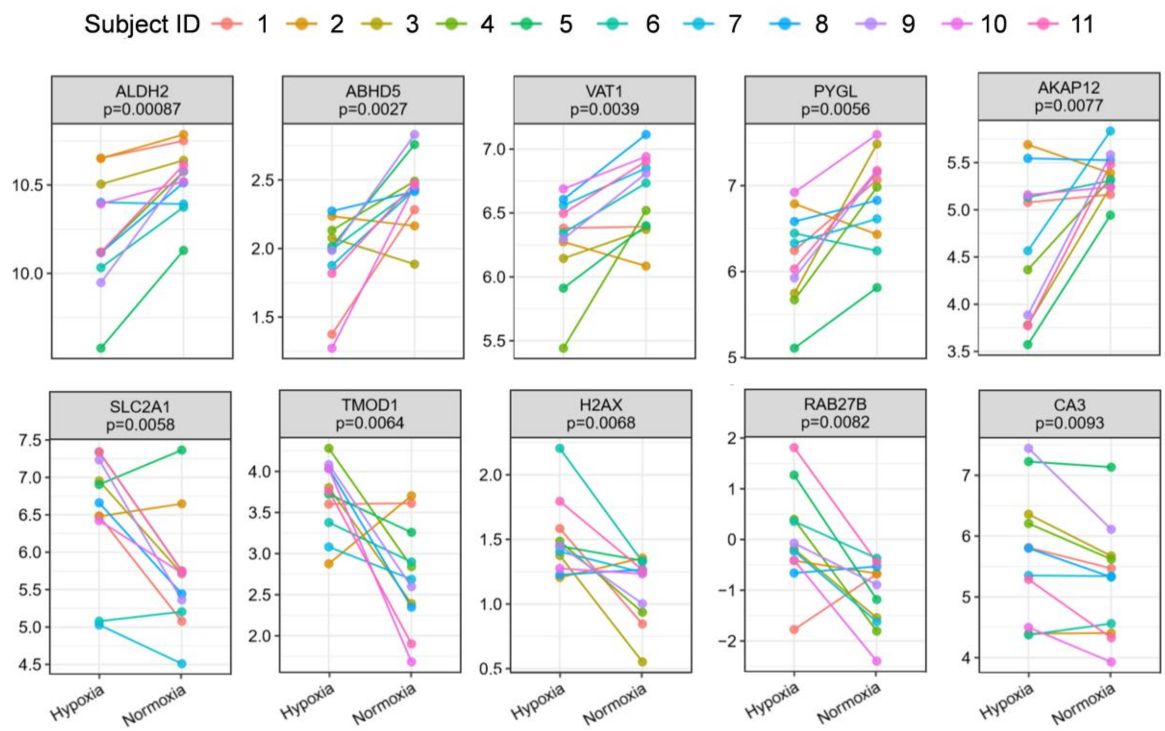

Supplementary Figure 1. Differentially expressed proteins in abdominal subcutaneous adipose tissue following MIH and normoxia exposure. Log2 protein expression is depicted for each individual separately (*n*=11), with corresponding *p*-value for top five up/downregulated (based on *p*-value) protein. ALDH2, aldehyde dehydrogenase 2; ABHD5, abhydrolase domain containing 5; VAT1, vesicle amine transport 1; PYGL, glycogen phosphorylase l; AKAP12, a-kinase anchoring protein 12; SLC2A1, solute carrier type 2A1/glucose transporter-1; TMOD1, tropomodulin-1; H2AX, H2A histone family member X; CA3, carbonic anhydrase III.

2 Supplementary Tables

Supplementary Table 1. Proteins exclusively expressed by blood and identified in human adipose tissue biopsies.

Category	Group	Uniprot	Name
Hemoglobins	Hemoglobins	P69905	Hemoglobin subunit alpha
Hemoglobins	Hemoglobins	P68871	Hemoglobin subunit beta
Hemoglobins	Hemoglobins	P69891	Hemoglobin subunit gamma-1
Hemoglobins	Hemoglobins	P69892	Hemoglobin subunit gamma-2
Hemoglobins	Hemoglobins	P02042	Hemoglobin subunit delta
Hemoglobins	Hemoglobins	P02008	Hemoglobin subunit zeta
Hemoglobins	Hemoglobins	P02100	Hemoglobin subunit epsilon
Hemoglobins	Hemoglobins	Q6B0K9	Hemoglobin subunit mu
Hemoglobins	Hemoglobins	P09105	Hemoglobin subunit theta-1
Serum albumin	Albumin	P02768	Serum albumin
Erythrocyte proteins	Erythrocyte	P02549	spectrin alpha chain, erythrocytic 1
Erythrocyte proteins	Erythrocyte	P11277	Spectrin beta chain, erythrocytic (Beta-I spectrin)
Erythrocyte proteins	Erythrocyte	P16157	Ankyrin-1
Erythrocyte proteins	Erythrocyte	P16452	Erythrocyte membrane protein band 4.2
Erythrocyte proteins	Erythrocyte	P11171	Erythrocyte membrane protein band 4.1
Erythrocyte proteins	Erythrocyte	Q00013	55 kDa erythrocyte membrane protein
Erythrocyte proteins	Erythrocyte	P27105	Erythrocyte band 7 integral membrane protein
Erythrocyte proteins	Erythrocyte	Q02094	Ammonium transporter Rh type A (Erythrocyte membrane glycoprotein Rh50)
Erythrocyte proteins	Erythrocyte	Q08495	Dematin (Dematin actin-binding protein) (Erythrocyte membrane protein band 4.9)
Other blood proteins	Prealbumins	P02766	Transthyretin
Other blood proteins	Prealbumins	P01009	Alpha-1-antitrypsin
Other blood proteins	Alpha 1 globulins	P02763	Alpha-1-acid glycoprotein 1
Other blood proteins	Alpha 1 globulins	P19652	Alpha-1-acid glycoprotein 2
Other blood proteins	Alpha 2 globulins	P00738	Haptoglobin
Other blood proteins	Alpha 2 globulins	P01023	α2-macroglobulin
Other blood proteins	Alpha 2 globulins	P00450	Ceruloplasmin
Other blood proteins	Beta globulins	P00747	plasminogen

Other blood proteins	Beta globulins	P02787	Serotransferrin
Other blood proteins	Immunoglobins	P01871	Immunoglobulin heavy constant mu
Other blood proteins	Immunoglobins	P01876	Immunoglobulin heavy constant alpha 1
Other blood proteins	Immunoglobins	P01877	Immunoglobulin heavy constant alpha 2
Other blood proteins	Immunoglobins	P01860	Immunoglobulin heavy constant gamma 3
Other blood proteins	Immunoglobins	P01764	Immunoglobulin heavy variable 3-23
Other blood proteins	Immunoglobins	P01857	Immunoglobulin heavy constant gamma 1
Other blood proteins	Immunoglobins	P01859	Immunoglobulin heavy constant gamma 2
Other blood proteins	Immunoglobins	P23083	Immunoglobulin heavy variable 1-2
Other blood proteins	Immunoglobins	P01861	Immunoglobulin heavy constant gamma 4
Other blood proteins	Immunoglobins	P01780	Immunoglobulin heavy variable 3-7
Other blood proteins	Immunoglobins	P01766	Immunoglobulin heavy variable 3-13
Other blood proteins	Immunoglobins	P01880	Immunoglobulin heavy constant delta
Other blood proteins	Immunoglobins	P01743	Immunoglobulin heavy variable 1-46
Other blood proteins	Immunoglobins	P06331	Immunoglobulin heavy variable 4-34
Other blood proteins	Immunoglobins	P01619	Immunoglobulin kappa variable 3-20
Other blood proteins	Immunoglobins	P01834	Immunoglobulin kappa constant
Other blood proteins	Immunoglobins	P01593	Immunoglobulin kappa variable 1D-33
Other blood proteins	Immunoglobins	P01602	Immunoglobulin kappa variable 1-5
Other blood proteins	Immunoglobins	P06312	Immunoglobulin kappa variable 4-1
Other blood proteins	Immunoglobins	P01611	Immunoglobulin kappa variable 1D-12
Other blood proteins	Immunoglobins	P04430	Immunoglobulin kappa variable 1-16
Other blood proteins	Immunoglobins	P06310	Immunoglobulin kappa variable 2-30
Other blood proteins	Immunoglobins	P04433	Immunoglobulin kappa variable 3-11
Other blood proteins	Immunoglobins	P01701	Immunoglobulin lambda variable 1-51
Other blood proteins	Immunoglobins	P01700	Immunoglobulin lambda variable 1-47
Other blood proteins	Immunoglobins	A0M8Q6	Immunoglobulin lambda constant 7
Other blood proteins	Immunoglobins	P80748	Immunoglobulin lambda variable 3-21
Other blood proteins	Immunoglobins	P01717	Immunoglobulin lambda variable 3-25
Other blood proteins	Immunoglobins	P01714	Immunoglobulin lambda variable 3-19
Other blood proteins	Immunoglobins	P01715	Immunoglobulin lambda variable 3-1
Other blood proteins	Immunoglobins	B9A064	Immunoglobulin lambda-like polypeptide 5
Other blood proteins	Immunoglobins	P04211	Immunoglobulin lambda variable 7-43
Other blood proteins	Immunoglobins	P0DOY2	Immunoglobulin lambda constant 2
Other blood proteins	Platelet	P02776	Platelet factor 4
Other blood proteins	Platelet	P13224	Platelet glycoprotein Ib beta chain

Supplementary Table 2. Proteins that were altered in adipose tissue by MIH compared to normoxia exposure in overweight and obese individuals at a significance level of p<0.05.

**Uniprot**	**Gene**	**Name**	**Fold change (MIH / Normoxia)**	***p*-value**
Q99715	COL12A1	Collagen alpha-1(XII) chain	4,07	0,041
Q13201	MMRN1	Multimerin-1	2,34	0,029
O00194	RAB27B	Ras-related protein Rab-27B	2,16	0,008
P80188	LCN2	Neutrophil gelatinase-associated lipocalin	2,04	0,018
P28289	TMOD1	Tropomodulin-1	1,97	0,006
P31146	CORO1A	Coronin-1A	1,93	0,018
P05106	ITGB3	Integrin beta-3	1,92	0,027
P13473	LAMP2	Lysosome-associated membrane glycoprotein 2	1,85	0,025
P11166	SLC2A1	Glucose transporter type 1	1,81	0,006
P41091	EIF2S3	Eukaryotic translation initiation factor 2 subunit 3	1,8	0,044
P02788	LTF	Lactotransferrin	1,66	0,049
P02730	SLC4A1	Solute carrier family 4 member 1	1,65	0,015
P29972	AQP1	Aquaporin-1	1,63	0,013
P09543	CNP	2',3'-cyclic-nucleotide 3'-phosphodiesterase	1,61	0,038
P54725	RAD23A	UV excision repair protein RAD23 homolog A	1,6	0,037
Q15942	ZYX	Zyxin	1,59	0,018
O60256	PRPSAP2	Phosphoribosyl pyrophosphate synthase-associated protein 2	1,5	0,018
P18577	RHCE	CD antigen CD240CE	1,49	0,029
P04843	RPN1	Ribophorin-1	1,48	0,03
O14561	NDUFAB1	Acyl carrier protein, mitochondrial	1,43	0,022
Q6XQN6	NAPRT	Nicotinate phosphoribosyltransferase	1,42	0,033
Q9ULV4	CORO1C	Coronin-1C	1,41	0,048
P07384	CAPN1	Calpain-1 catalytic subunit	1,39	0,013
P21333	FLNA	Filamin-A	1,38	0,015
P52566	ARHGDIB	Rho GDP-dissociation inhibitor	1,38	0,037
P07451	CA3	Carbonic anhydrase 3	1,36	0,009
P04439	HLA-A	HLA class I histocompatibility antigen, A alpha chain	1,36	0,035
Q9NYL9	TMOD3	Tropomodulin-3	1,32	0,039
O15511	ARPC5	Actin-related protein 2/3 complex subunit 5	1,3	0,015
P16104	H2AX	Histone H2AX	1,29	0,007
P67936	TPM4	Tropomyosin alpha-4 chain	1,27	0,047

P54920	NAPA	Alpha-soluble NSF attachment protein	1,26	0,025
P07737	PFN1	Profilin-1	1,25	0,027
P06753	TPM3	Tropomyosin alpha-3 chain	1,24	0,043
Q9H773	DCTPP1	dCTP pyrophosphatase 1	1,23	0,043
P61026	RAB10	Ras-related protein Rab-10	1,23	0,027
P09493	TPM1	Tropomyosin alpha-1 chain	1,21	0,016
P22061	PCMT1	Protein-L-isoaspartate(D-aspartate) O-methyltransferase	1,21	0,015
P26447	S100A4	Protein S100-A4	1,2	0,05
P30626	SRI	Sorcin	1,17	0,022
P48643	CCT5	T-complex protein 1 subunit epsilon	1,15	0,021
P50395	GDI2	Rab GDP dissociation inhibitor beta	1,09	0,026
P0DMV8	HSPA1A	Heat shock 70 kDa protein 1A	0,88	0,022
P49368	CCT3	T-complex protein 1 subunit gamma	0,87	0,026
P05091	ALDH2	Aldehyde dehydrogenase, mitochondrial	0,81	0,001
P61981	YWHAG	14-3-3 protein gamma	0,8	0,047
P00338	LDHA	L-lactate dehydrogenase A chain	0,8	0,046
Q9NWV4	CZIB	CXXC motif containing zinc binding protein	0,79	0,032
P11047	LAMC1	Laminin subunit gamma-1	0,78	0,049
Q99536	VAT1	Synaptic vesicle membrane protein VAT-1 homolog	0,78	0,004
P99999	CYCS	Cytochrome c	0,77	0,04
P98160	HSPG2	Basement membrane-specific heparan sulfate proteoglycan core protein	0,76	0,041
P07355	ANXA2	Annexin A2	0,76	0,036
P13671	C6	Complement component C6	0,76	0,023
Q9HBL0	TNS1	Tensin-1	0,75	0,035
P19827	ITIH1	Inter-alpha-trypsin inhibitor heavy chain H1	0,75	0,043
P01008	SERPINC1	Antithrombin-III	0,75	0,037
P16403	H1-2	Histone H1.2	0,73	0,009
Q96Q06	PLIN4	Perilipin-4	0,72	0,036
Q13813	SPTAN1	Spectrin alpha chain, non-erythrocytic 1	0,72	0,039
P49327	FASN	Fatty acid synthase	0,7	0,049
P07585	DCN	Decorin	0,7	0,046
P25311	AZGP1	Zinc-alpha-2-glycoprotein	0,7	0,04
P55268	LAMB2	Laminin subunit beta-2	0,7	0,026
Q8WTS1	ABHD5	1-acylglycerol-3-phosphate O-acyltransferase ABHD5	0,7	0,003
Q02218	OGDH	2-oxoglutarate dehydrogenase, mitochondrial	0,7	0,044
P49189	ALDH9A1	Aldehyde dehydrogenase family 9 member A1	0,69	0,022

P59768	GNG2	Guanine nucleotide-binding protein G(I)/G(S)/G(O) subunit gamma-2	0,69	0,024
P43686	PSMC4	26S proteasome regulatory subunit 6B	0,68	0,049
P22676	CALB2	Calretinin	0,68	0,049
Q9BRX8	PRXL2A	Peroxiredoxin-like 2A	0,68	0,044
P22352	GPX3	Glutathione peroxidase 3	0,68	0,041
P09936	UCHL1	Ubiquitin carboxyl-terminal hydrolase isozyme L1	0,67	0,044
Q16363	LAMA4	Laminin subunit alpha-4	0,66	0,034
Q13642	FHL1	Four and a half LIM domains protein 1	0,66	0,043
Q6NZI2	CAVIN1	Caveolae-associated protein 1	0,66	0,043
O60240	PLIN1	Perilipin-1	0,65	0,019
Q07507	DPT	Dermatopontin	0,65	0,026
Q16851	UGP2	UDP-glucose pyrophosphorylase	0,65	0,008
Q9UHG3	PCYOX1	Prenylcysteine oxidase 1	0,64	0,037
P50238	CRIP1	Cysteine-rich protein 1	0,64	0,039
Q9NZN4	EHD2	EH domain-containing protein 2	0,64	0,026
Q16555	DPYSL2	Dihydropyrimidinase-related protein 2	0,64	0,024
P14543	NID1	Nidogen-1	0,64	0,045
O00159	MYO1C	Unconventional myosin-Ic	0,64	0,022
P07099	EPHX1	Epoxide hydrolase 1	0,64	0,014
P15090	FABP4	Fatty acid-binding protein 4	0,63	0,034
Q12797	ASPH	Aspartyl/asparaginyl beta-hydroxylase	0,62	0,046
P06737	PYGL	Glycogen phosphorylase, liver form	0,62	0,006
P10301	RRAS	Ras-related protein R-Ras	0,61	0,017
O75874	IDH1	Isocitrate dehydrogenase [NADP] cytoplasmic	0,61	0,033
P21397	MAOA	Monoamine oxidase type A	0,61	0,045
Q04828	AKR1C1	Aldo-keto reductase family 1 member C1	0,6	0,047
Q14112	NID2	Nidogen-2	0,6	0,019
Q02952	AKAP12	A-kinase anchor protein 12	0,59	0,008
P49753	ACOT2	Acyl-coenzyme A thioesterase 2	0,59	0,038
P02511	CRYAB	Alpha-crystallin B chain	0,58	0,017
P19105	MYL12A	Myosin regulatory light chain 12A	0,58	0,033
P15559	NQO1	NAD(P)H dehydrogenase [quinone] 1	0,58	0,022
P11766	ADH5	Alcohol dehydrogenase class-3	0,57	0,025
P52895	AKR1C2	Aldo-keto reductase family 1 member C2	0,57	0,024
P21695	GPD1	Glycerol-3-phosphate dehydrogenase [NAD(+)], cytoplasmic	0,56	0,016
P30084	ECHS1	Enoyl-CoA hydratase, mitochondrial	0,56	0,027
Q05469	LIPE	Hormone-sensitive lipase	0,56	0,027
P09417	QDPR	Dihydropteridine reductase	0,55	0,034

P10620	MGST1	Microsomal glutathione S-transferase 1	0,55	0,042
P36543	ATP6V1E1	V-type proton ATPase subunit E 1	0,54	0,026
P00325	ADH1B	Alcohol dehydrogenase 1B	0,54	0,021
P60903	S100A10	Protein S100-A10	0,54	0,022
Q9BX66	SORBS1	Sorbin and SH3 domain-containing protein 1	0,53	0,019
P46939	UTRN	Utrophin	0,52	0,047
Q9NVD7	PARVA	Alpha-parvin	0,51	0,015
Q16836	HADH	Hydroxyacyl-coenzyme A dehydrogenase, mitochondrial	0,51	0,018
P54819	AK2	Adenylate kinase 2, mitochondrial	0,51	0,045
P55809	OXCT1	Succinyl-CoA:3-ketoacid coenzyme A transferase 1, mitochondrial	0,5	0,034
P36871	PGM1	Phosphoglucomutase-1	0,48	0,02
P48047	ATP5PO	ATP synthase subunit O, mitochondrial	0,47	0,041
P08294	SOD3	Extracellular superoxide dismutase	0,47	0,02
Q9BX68	HINT2	Histidine triad nucleotide-binding protein 2, mitochondrial	0,46	0,021
P22105	TNXB	Tenascin-X	0,44	0,022
O76070	SNCG	Gamma-synuclein	0,43	0,025
P11177	PDHB	Pyruvate dehydrogenase E1 component subunit beta, mitochondrial	0,42	0,03
P16083	NQO2	Ribosyldihydronicotinamide dehydrogenase [quinone]	0,33	0,014

Supplementary Table 3. Functional groups affected by mild intermittent hypoxia exposure. After functional annotation of differentially expressed proteins, 11 functional groups were identified ($p<0.05$, corrected with Bonferroni step-down procedure) covering 104 GO terms and KEGG pathways, which were altered by mild intermittent hypoxia exposure. *p*-values are corrected with Bonferroni step-down procedure. GO, Gene Ontology; KEGG, Kyoto Encyclopedia of Genes and Genomes.

**Functional group**	**GO Term**	**GO ID**	**GO Term *p*-value**	**Functional group *p*-value**
cellular iron ion homeostasis	cellular iron ion homeostasis	GO:0006879	5,86E-02	1,17E-02
bicarbonate transport	bicarbonate transport	GO:0015701	9,01E-02	9,66E-03
platelet degranulation	platelet degranulation	GO:0002576	8,23E-02	1,03E-02
collagen fibril organization	collagen fibril organization	GO:0030199	2,41E-02	3,05E-03
myeloid cell development	myeloid cell development	GO:0061515	7,39E-02	9,48E-03
Regulation of lipolysis in adipocytes	Regulation of lipolysis in adipocytes	KEGG:04923	3,23E-02	1,03E-02
	PPAR signaling pathway	KEGG:03320	7,23E-02	1,03E-02
	neutral lipid catabolic process	GO:0046461	9,61E-02	1,03E-02
	glycerolipid catabolic process	GO:0046503	4,39E-02	1,03E-02
	acylglycerol catabolic process	GO:0046464	9,61E-02	1,03E-02
	triglyceride catabolic process	GO:0019433	7,43E-02	1,03E-02
ADP metabolic process	ADP metabolic process	GO:0046031	2,38E-02	4,91E-03
	purine nucleoside diphosphate metabolic process	GO:0009135	3,68E-02	4,91E-03
	ribonucleoside diphosphate metabolic process	GO:0009185	3,92E-02	4,91E-03
	ATP generation from ADP	GO:0006757	7,41E-02	4,91E-03
	purine ribonucleoside diphosphate metabolic process	GO:0009179	3,68E-02	4,91E-03
	glycolytic process	GO:0006096	7,36E-02	4,91E-03
polysaccharide biosynthetic process	polysaccharide biosynthetic process	GO:0000271	2,03E-02	1,23E-02
	Starch and sucrose metabolism	KEGG:00500	7,40E-02	1,23E-02
	polysaccharide metabolic process	GO:0005976	8,86E-02	1,23E-02
	energy reserve metabolic process	GO:0006112	9,66E-02	1,23E-02
	cellular carbohydrate biosynthetic process	GO:0034637	9,96E-02	1,23E-02

	cellular polysaccharide metabolic process	GO:0044264	1,00E-01	1,23E-02
	glucan metabolic process	GO:0044042	8,21E-02	1,23E-02
	cellular glucan metabolic process	GO:0006073	8,21E-02	1,23E-02
	cellular polysaccharide biosynthetic process	GO:0033692	3,24E-02	1,23E-02
	glycogen metabolic process	GO:0005977	8,09E-02	1,23E-02
	glucan biosynthetic process	GO:0009250	1,04E-01	1,23E-02
	glycogen biosynthetic process	GO:0005978	1,04E-01	1,23E-02
Actin cytoskeleton organization	substrate adhesion-dependent cell spreading	GO:0034446	7,37E-04	1,04E-06
	Cardiac muscle contraction	KEGG:04260	8,47E-02	1,04E-06
	Hypertrophic cardiomyopathy (HCM)	KEGG:05410	9,26E-02	1,04E-06
	Dilated cardiomyopathy (DCM)	KEGG:05414	9,66E-02	1,04E-06
	actin-mediated cell contraction	GO:0070252	1,59E-03	1,04E-06
	muscle cell migration	GO:0014812	8,98E-02	1,04E-06
	actin-myosin filament sliding	GO:0033275	9,36E-03	1,04E-06
	regulation of ATPase activity	GO:0043462	8,21E-02	1,04E-06
	smooth muscle cell migration	GO:0014909	7,05E-02	1,04E-06
	muscle filament sliding	GO:0030049	9,36E-03	1,04E-06
	myofibril assembly	GO:0030239	6,77E-02	1,04E-06
	ruffle organization	GO:0031529	1,05E-01	1,04E-06
	positive regulation of ATPase activity	GO:0032781	9,55E-02	1,04E-06
	negative regulation of protein polymerization	GO:0032272	7,28E-02	1,04E-06
	positive regulation of actin filament bundle assembly	GO:0032233	5,18E-02	1,04E-06
	negative regulation of actin filament polymerization	GO:0030837	4,63E-02	1,04E-06
Focal adhesion	Focal adhesion	KEGG:04510	6,87E-04	2,80E-09
	ECM-receptor interaction	KEGG:04512	2,27E-03	2,80E-09
	Prion diseases	KEGG:05020	6,98E-02	2,80E-09
	Toxoplasmosis	KEGG:05145	4,80E-02	2,80E-09
	Small cell lung cancer	KEGG:05222	8,98E-02	2,80E-09
	muscle cell migration	GO:0014812	8,98E-02	2,80E-09
	substrate adhesion-dependent cell spreading	GO:0034446	7,37E-04	2,80E-09
	cell-substrate junction organization	GO:0150115	9,14E-03	2,80E-09
	smooth muscle cell migration	GO:0014909	7,05E-02	2,80E-09

	extracellular matrix assembly	GO:0085029	9,27E-02	2,80E-09
	positive regulation of cell-matrix adhesion	GO:0001954	9,23E-02	2,80E-09
	cell-substrate junction assembly	GO:0007044	7,25E-03	2,80E-09
	neuromuscular junction development	GO:0007528	1,14E-01	2,80E-09
	basement membrane organization	GO:0071711	5,08E-03	2,80E-09
	glomerulus development	GO:0032835	8,67E-02	2,80E-09
	glomerular basement membrane development	GO:0032836	4,82E-03	2,80E-09
	positive regulation of actin filament bundle assembly	GO:0032233	5,18E-02	2,80E-09
Oxidoreductase activity	oxidoreductase activity, acting on the CH-OH group of donors, NAD or NADP as acceptor	GO:0016616	2,72E-05	4,67E-10
	oxidoreductase activity, acting on the aldehyde or oxo group of donors, NAD or NADP as acceptor	GO:0016620	5,95E-04	4,67E-10
	oxidoreductase activity, acting on the CH-CH group of donors, NAD or NADP as acceptor	GO:0016628	3,93E-02	4,67E-10
	oxidoreductase activity, acting on NAD(P)H, quinone or similar compound as acceptor	GO:0016655	4,90E-03	4,67E-10
	tricarboxylic acid cycle	GO:0006099	8,73E-02	4,67E-10
	ethanol metabolic process	GO:0006067	2,54E-02	4,67E-10
	aerobic respiration	GO:0009060	9,96E-02	4,67E-10
	aldehyde dehydrogenase [NAD(P)+] activity	GO:0004030	1,28E-02	4,67E-10
	diterpenoid metabolic process	GO:0016101	7,68E-02	4,67E-10
	aldehyde dehydrogenase (NAD+) activity	GO:0004029	9,05E-03	4,67E-10
	acyl-CoA metabolic process	GO:0006637	1,11E-01	4,67E-10
	retinoid metabolic process	GO:0001523	9,96E-02	4,67E-10
	Glycolysis / Gluconeogenesis	KEGG:00010	3,23E-05	4,67E-10
	Citrate cycle (TCA cycle)	KEGG:00020	5,18E-02	4,67E-10
	Fatty acid elongation	KEGG:00062	4,09E-02	4,67E-10
	Fatty acid degradation	KEGG:00071	4,13E-05	4,67E-10
	Valine, leucine and isoleucine degradation	KEGG:00280	1,42E-03	4,67E-10

	Lysine degradation	KEGG:00310	4,09E-02	4,67E-10
	Arginine and proline metabolism	KEGG:00330	9,72E-02	4,67E-10
	Histidine metabolism	KEGG:00340	2,86E-02	4,67E-10
	Tyrosine metabolism	KEGG:00350	7,40E-02	4,67E-10
	Tryptophan metabolism	KEGG:00380	7,44E-04	4,67E-10
	beta-Alanine metabolism	KEGG:00410	5,47E-02	4,67E-10
	Glutathione metabolism	KEGG:00480	1,07E-01	4,67E-10
	Pyruvate metabolism	KEGG:00620	9,36E-03	4,67E-10
	Butanoate metabolism	KEGG:00650	4,48E-02	4,67E-10
	Metabolism of xenobiotics by cytochrome P450	KEGG:00980	1,18E-02	4,67E-10
	Drug metabolism	KEGG:00982	6,21E-02	4,67E-10
	Chemical carcinogenesis	KEGG:05204	1,60E-02	4,67E-10
	Central carbon metabolism in cancer	KEGG:05230	5,42E-02	4,67E-10
	detoxification	GO:0098754	3,92E-02	4,67E-10
	electron transport chain	GO:0022900	4,94E-03	4,67E-10
	thioester metabolic process	GO:0035383	1,11E-01	4,67E-10
	cellular detoxification	GO:1990748	1,61E-02	4,67E-10
	xenobiotic metabolic process	GO:0006805	8,23E-02	4,67E-10
	oxidoreductase activity, acting on CH-OH group of donors	GO:0016614	4,31E-05	4,67E-10
	oxidoreductase activity, acting on the CH-CH group of donors	GO:0016627	9,19E-02	4,67E-10
	oxidoreductase activity, acting on the aldehyde or oxo group of donors	GO:0016903	3,76E-03	4,67E-10
	antibiotic metabolic process	GO:0016999	2,19E-02	4,67E-10
	drug catabolic process	GO:0042737	9,84E-02	4,67E-10
	electron transfer activity	GO:0009055	1,40E-02	4,67E-10
	antibiotic catabolic process	GO:0017001	5,06E-02	4,67E-10
	primary alcohol metabolic process	GO:0034308	4,07E-03	4,67E-10
	ethanol oxidation	GO:0006069	4,82E-03	4,67E-10
